# Preconditioning with Endoplasmic Reticulum Stress Ameliorates Endothelial Cell Inflammation

**DOI:** 10.1371/journal.pone.0110949

**Published:** 2014-10-30

**Authors:** Antony Leonard, Adrienne W. Paton, Monaliza El-Quadi, James C. Paton, Fabeha Fazal

**Affiliations:** 1 Department of Pediatrics, Lung Biology and Disease Program, University of Rochester School of Medicine and Dentistry, Rochester, New York, United States of America; 2 Research Centre for Infectious Diseases, School of Molecular and Biomedical Science, University of Adelaide, Adelaide, South Australia, Australia; University of Illinois at Chicago, United States of America

## Abstract

Endoplasmic Reticulum (ER) stress, caused by disturbance in ER homeostasis, has been implicated in several pathological conditions such as ischemic injury, neurodegenerative disorders, metabolic diseases and more recently in inflammatory conditions. Our present study aims at understanding the role of ER stress in endothelial cell (EC) inflammation, a critical event in the pathogenesis of acute lung injury (ALI). We found that preconditioning human pulmonary artery endothelial cells (HPAEC) to ER stress either by depleting ER chaperone and signaling regulator BiP using siRNA, or specifically cleaving (inactivating) BiP using subtilase cytotoxin (SubAB), alleviates EC inflammation. The two approaches adopted to abrogate BiP function induced ATF4 protein expression and the phosphorylation of eIF2α, both markers of ER stress, which in turn resulted in blunting the activation of NF-κB, and restoring endothelial barrier integrity. Pretreatment of HPAEC with BiP siRNA inhibited thrombin-induced IκBα degradation and its resulting downstream signaling pathway involving NF-κB nuclear translocation, DNA binding, phosphorylation at serine536, transcriptional activation and subsequent expression of adhesion molecules. However, TNFα-mediated NF-κB signaling was unaffected upon BiP knockdown. In an alternative approach, SubAB-mediated inactivation of NF-κB was independent of IκBα degradation. Mechanistic analysis revealed that pretreatment of EC with SubAB interfered with the binding of the liberated NF-κB to the DNA, thereby resulting in reduced expression of adhesion molecules, cytokines and chemokines. In addition, both knockdown and inactivation of BiP stimulated actin cytoskeletal reorganization resulting in restoration of endothelial permeability. Together our studies indicate that BiP plays a central role in EC inflammation and injury via its action on NF-κB activation and regulation of vascular permeability.

## Introduction

Endoplasmic reticulum, an intricate cellular organelle present in eukaryotic cells, is a major site for the synthesis and maturation of secretory and membrane proteins [1]–[3]. Protein synthesis in ER is dynamically regulated as per the physiological need of the cell. However, a wide variety of disturbances such as glucose deprivation, changes in redox status, disruption of calcium homeostasis and viral and bacterial infections can cause imbalance in the luminal flux of the newly synthesized unfolded or misfolded peptides resulting in a condition known as ER stress. To combat ER stress, an evolutionarily conserved adaptive mechanism, termed the unfolded protein response (UPR) is activated and assists in cell survival. However, if the ER dysfunction is prolonged and severe, the UPR initiates cell death via apoptosis or autophagy. A key component involved in the regulation and activation of the UPR is the ER chaperone BiP (Binding Immunoglobulin Protein), a 78-kDa glucose-regulated protein (GRP78), also referred to as heat-shock protein A5 (HSPA5). In the unstressed state, BiP is found associated with the luminal domains of three ER stress sensors, protein kinase RNA-like ER kinase (PERK), inositol-requiring enzyme (IRE)1-α/β and activating transcription factor (ATF)6-α/β. However, upon induction of ER stress, BiP dissociates from the ER signal sensors, causing their phosphorylation, activation and translocation. Together, these three branches of the UPR restore ER homeostasis [4], [5]. ER stress is mechanistically linked to inflammation at several levels [6] as evidenced by the fact that it is an underlying factor in the pathogenesis of several metabolic and immunological diseases, with inflammatory underpinning, such as obesity, diabetes, inflammatory bowel disease (IBD) and glomerular disease [7]–[10].

Inflammation is the body’s protective mechanism against infection or injury and it tends to resolve once the source has been cleared from the system. However, when the inflammatory response becomes severe or prolonged it results in a diseased state such as acute lung injury (ALI). This is characterized by massive infiltration of polymorphonuclear leukocytes (PMN) from the blood into the lung that leads to disruption of vascular endothelial permeability and development of pulmonary edema, with severe consequences for pulmonary gas exchange [11]–[17]. The movement of PMN from the blood to the inflammatory site involves the interaction between intercellular cell adhesion molecule-1 (ICAM-1) on EC surfaces and its counter receptor β2 integrins (CD11/CD18) on the surface of PMNs (18). Chemokines Interleukin-8 (IL-8) and monocyte chemotactic protein-1 (MCP-1) are immune mediators involved in targeting leukocytes and monocytes to sites of inflammation. The expression of ICAM-1, VCAM-1, IL-8 and MCP-1 is under the tight control of the inflammatory transcription factor NF-κB [18], [19]. NF-κB is activated upon phosphorylation of its cytoplasmic inhibitor IκBα on Serine^32^ and Serine^36^ by IκB kinase (IKK) complex. Phosphorylation triggers the ubiquitination-mediated degradation of IκBα, which results in nuclear translocation of NF-κB and subsequent transcription of inflammatory genes.

Interestingly, recent studies have also shown that ER stress regulates NF-κB activity in a biphasic and bidirectional manner [7] in different cell types, contributing to the pathogenesis of diseases such as cancer, amyotrophic lateral sclerosis (ALS) and diabetic retinopathy [20]–[23]. In contrast, studies have also shown that ER stress preconditioning protects the cells against a number of inflammatory stimuli [24]–[30]. Deregulated NFκB activity has been implicated in a wide range of human diseases including cancer, ALI, diabetes, arthritis, and infection [31]–[35].

In the present study we adopted a dual approach to unravel the role of the ER stress regulatory protein BiP in EC inflammation and injury associated with ALI. Our data show that preconditioning the endothelial cells with ER stress by depleting BiP using siRNA, or by inactivating BiP using SubAB [36], mitigates inflammation by blunting the NF-κB transcriptional activity and by restoring vascular endothelial permeability.

## Materials and Methods

### Reagents

Human thrombin was obtained from Enzyme Research Laboratories (South Bend, IN). Tunicamycin was purchased from Sigma. Polyclonal antibodies to ICAM-1, VCAM-1, RelA/p65, β-actin, IκBα, and Lamin B were from Santa Cruz Biotechnology (Santa Cruz, CA). Antibodies to phospho-eIF2α, eIF2α, BiP/GRP78, ATF-4, phospho-AKT, phospho-IKKβ, IKKβ, phospho-(Ser^536^)-RelA/p65, were obtained from Cell Signaling (Beverly, MA). Tata Binding Protein (TBP) antibody was purchased from Abcam. RelA/p65 transcription factor assay kit was purchased from Cayman Chemical (Ann Arbor, MI) and plasmid maxi kit was from QIAGEN Inc. (Valencia, CA). Protein assay kit and nitrocellulose membrane were from Bio-Rad. Alexa Fluor 488-phalloidin was purchased from Invitrogen. Expression vector encoding Wild type BiP and dominant negative BiP were from Addgene. All other materials were from VWR Scientific Products Corporation (Gaithersburg, MD) and Fisher Scientific (Pittsburgh, PA). SubAB and its non-toxic derivative SubA_A272_B were purified as previously described [37].

### Cell Culture

Human pulmonary artery endothelial cells (HPAEC) were purchased from Lonza (Walkersville, MD). Cells were cultured as described previously [38], [39] in endothelial basal medium 2 (EBM2) supplemented with bullet kit additives (Lonza, Walkersville, MD) and were used between passages 3 and 7.

### RNAi knockdown

SMARTpool siRNA specific for human BiP and a non-targeting siRNA control were purchased from Dharmacon (Lafayette, CO). EC were transfected with BiP siRNA or control siRNA using DharmaFect1 siRNA Transfection Reagent (Dharmacon) essentially as described [40].

### NF-κB Transcriptional Activity

The construct pNF-κB-LUC containing five copies of consensus NF-κB sequences linked to a minimal E1B promoter-luciferase gene was purchased from Stratagene (La Jolla, CA). Transfections were performed using the DEAE-dextran method essentially as described [41]. Briefly, 5 µg of DNA was mixed with 50 µg/ml DEAE-dextran in serum-free endothelial basal medium 2, and the mixture was added onto plasmid (Promega, Madison, WI) containing *Renilla* luciferase gene driven by the constitutively active thymidine kinase promoter to normalize transfection efficiencies. After 1 h, the cells were incubated for 4 min with 10% dimethyl sulfoxide in serum-free endothelial basal medium 2. The cells were then washed two times with endothelial basal medium 2, 10% fetal bovine serum and grown to confluence. We achieved transfection efficiency of 16±3 (mean ± S.D.; *n* = 3) in these cells. Cell extracts were prepared and assayed for firefly luciferase activity using the Promega Biotech dual luciferase reporter assay system. The data were expressed as a ratio of firefly luciferase activity. For experiments examining the effect of BiP knockdown on NF-κB activity, the cells were first transfected with siRNA using DharmaFect1. After 12–16 h, the cells were again transfected with pNF-κBLUC using the DEAE-dextran method, and luciferase activity was determined as described above. cells that were 60–80% confluent. We used 0.125 µg of pTKRLUC.

### Immunoblot Analysis

EC were lysed in radioimmune precipitation (RIPA) buffer containing 50 mM Tris-HCl, pH 7.4, 150 mM NaCl, 0.25 mM EDTA, pH 8.0, 1% deoxycholic acid, 1% Triton X-100, 5 mM NaF, 1 mM sodium orthovanadate supplemented with complete protease inhibitors (Sigma). The residual binding sites on the filters were blocked by incubating with 5% (w/v) nonfat dry milk in TBST (10 mM Tris, pH 8.0, 150 mM NaCl, 0.05% Tween 20) or 5% BSA in TBST for 1 h at room temperature. The membranes were subsequently incubated with the indicated antibodies and developed using an enhanced chemiluminescence (ECL) method, as described [42]. Representative blots shown in the result section come from the same membrane which may have more samples in various groups.

### ELISA

The levels of IL-8 and MCP-1 in HPAEC culture supernatants were determined using ELISA kits from R&D Systems (Minneapolis, MN) according to the manufacturer’s recommendations.

### Nuclear Extract Preparation and Assessment of RelA/p65 DNA Binding

After treatment, cells were washed twice with ice-cold phosphate-buffered saline and resuspended in 400 µl of buffer A (10 mM HEPES [pH 7.9], 10 mM KCl, 0.1 mM EDTA, 0.1 mM EGTA, 1 mM [DTT], and 0.5 mM PMSF). Fifteen minutes later, NP-40 was added to a final concentration of 0.6%, and the samples were centrifuged to collect the supernatants containing the cytoplasmic proteins. The pelleted nuclei were resuspended in 50 µl of buffer B (20 mM HEPES [pH 7.9], 0.4 M NaCl, 1 mM EDTA, 1 mM EGTA, 1 mM DTT, and 1 mM PMSF). After 0.5 h at 4°C, lysates were centrifuged and supernatants containing the nuclear proteins were collected. The DNA binding activity of RelA/p65 was determined using an ELISA-based DNA binding assay kit (Cayman Chemical, Ann Arbor, MI) in accordance with the manufacturer’s recommendations.

### Immunofluorescence

Cells grown on Collagen I coated coverslips were fixed in 3.7% paraformaldehyde/PBS for 10 min at room temperature (RT) and then permeabilized with 0.1% Triton X-100 for 5 min at room temperature as described [38], [43]. Permeabilized cells were rinsed 3 times with 1XPBS and incubated in blocking solution (5% normal goat serum or 1% bovine serum albumin/PBS) for 1 h at room temperature to prevent nonspecific binding of the antibody. All subsequent steps were carried out at room temperature and cells were rinsed 3 times with 1xPBS between each of the steps. To localize Filamentous-actin (F-actin), cells were incubated with Alexa Flour 488-labeled phalloidin for 20 min at room temperature. The coverslips were rinsed in PBS and mounted on the slide using Vectashield mounting media (Vector Laboratories, Lincolnshire, IL). Images were obtained using Nikon Eclipse - TE2000-E Fluorescent microscope and Olympus FV1000 confocal microscope.

### Electrophoretic Mobility Shift Assay (EMSA)

The EMSA was performed basically as described [44]. Briefly, 10 µg of nuclear extract was mixed with 1 µg of poly (dI-dC) in a binding buffer (10 mM Tris-HCl [pH 7.5], 50 mM NaCl, 0.5 mM DTT, 10% glycerol (20 µl final volume) for 15 min at room temperature. The double stranded oligonucleotides were end labeled using gamma^32^P ATP. The reaction mixture was incubated with the end-labeled double-stranded oligonucleotides containing an NF-κB site (30,000 cpm each) for 15 min at room temperature. The DNA-protein complexes were resolved on a 5% native polyacrylamide gel in low ionic strength buffer (0.25x Tris-borate-EDTA). The oligonucleotide used for the gel shift analysis was Ig-κB 5′-GTTGAGGGGACTTTCCCAGGC-3′ and contains the consensus NF-κB binding site sequence (underlined) present in mouse Ig kappa light chain gene [45].

### Endothelial permeability assay

Endothelial permeability was measured using Millipore’s *In vitro* Vascular Permeability Assay Kit. HPAEC transfected with control-siRNA or BiP-siRNA for 48 hours or treated with SubAB or SubA_A272_B for 6 hours were seeded at 20,000 cells per transwell insert and cultured for 48 hours. Following this, the confluent monolayer was treated with thrombin (5 U/ml) for 30 minutes. FITC-Dextran permeability testing was done to check monolayer integrity. Permeation was stopped by removing the inserts from the wells. Media from the receiver tray was transferred to a 96 well opaque plate to measure fluorescence. Fluorescent intensities were quantified using a fluorescent plate reader with filters appropriate for 485 nm and 535 nm excitation and emission. The endothelial monolayer was stained to check for monolayer integrity using Cell Stain provided in the kit. Cells were visualized using the Leica DMI 3000B fluorescent microscope.

### Statistical Analysis

Results are presented as mean ± SE and were analyzed by using standard one-way ANOVA. The significance between the groups was determined using Tukey’s test (Prism 5.0, GraphPad Software, San Diego). A p value<0.05 between two groups was considered statistically significant.

## Results

### RNAi knockdown of BiP induces eIF2α phosphorylation and ATF4 protein expression

**Figure 1:**
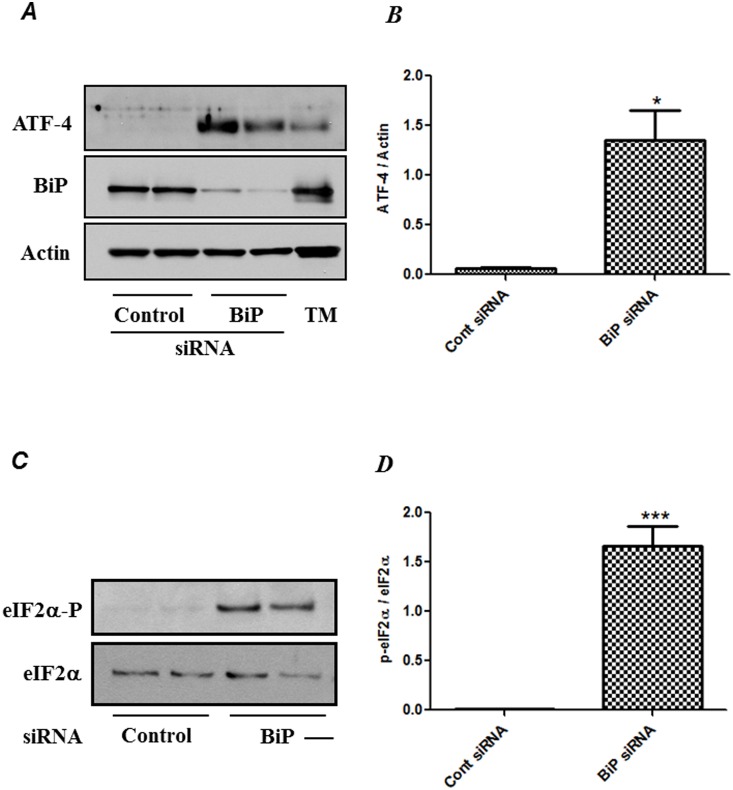
BiP knockdown induces ER stress. HPAEC were transfected with control siRNA or BiP siRNA for 48 h using DharmaFect1. Total cell lysates were separated by SDS-PAGE and immunoblotted with *(* ***A*** *)* anti-ATF4 antibody, and anti-actin antibody were used to monitor loading and anti-BiP antibody was used to monitor depletion and with *(* ***C*** *)* anti-phospho-eIF2α and anti-eIF2α antibody. TM, tunicamycin was used as a positive control. The data are the means ± S.E. (n = 3–6 for each condition). The bar graph represents the effect of BiP depletion on *(* ***B*** *)* ATF-4 expression and *(* ***D*** *)* eIF2-α phosphorylation, normalized to actin level. The data are the means ± S.E. (n = 3–6 for each condition). *p<0.01 or ***p<0.001 difference from controls.

To understand the significance of ER stress in EC inflammation, we first determined whether siRNA-mediated knockdown of BiP induces ER stress. ER stress activates a signaling network called the unfolded protein response (UPR) to alleviate the stress and promote cell survival. Of the three distinct signaling pathways that are activated by UPR, one of the pathways involves PERK autophosphorylation and dimerization, leading to phosphorylation of eIF2α, and concurrent increase in the expression of ATF4 that regulates the transcription of several UPR target genes that mitigate ER stress. Therefore, we investigated whether BiP knockdown induces the phosphorylation of eIF2α and ATF4 protein levels, the two markers of ER stress. HPAEC were transfected with control-siRNA or BiP-siRNA for 48 hrs and were then subjected to Western blot analysis. Results showed that BiP expression was significantly inhibited in cells transfected with BiP siRNA compared to the control siRNA and that BiP-depleted cells showed a robust increase in ATF4 protein expression (Fig. 1A & B). Also, cells transfected with BiP siRNA showed a marked induction in the phosphorylation of eIF2α (Fig. 1C & D). Tunicamycin (TM) was used as a positive control to monitor ER stress (Fig. 1A). These results indicate the induction of ER stress upon BiP knockdown.

### RNAi knockdown of BiP inhibits thrombin-induced NF-κB activity and adhesion molecules expression

**Figure 2:**
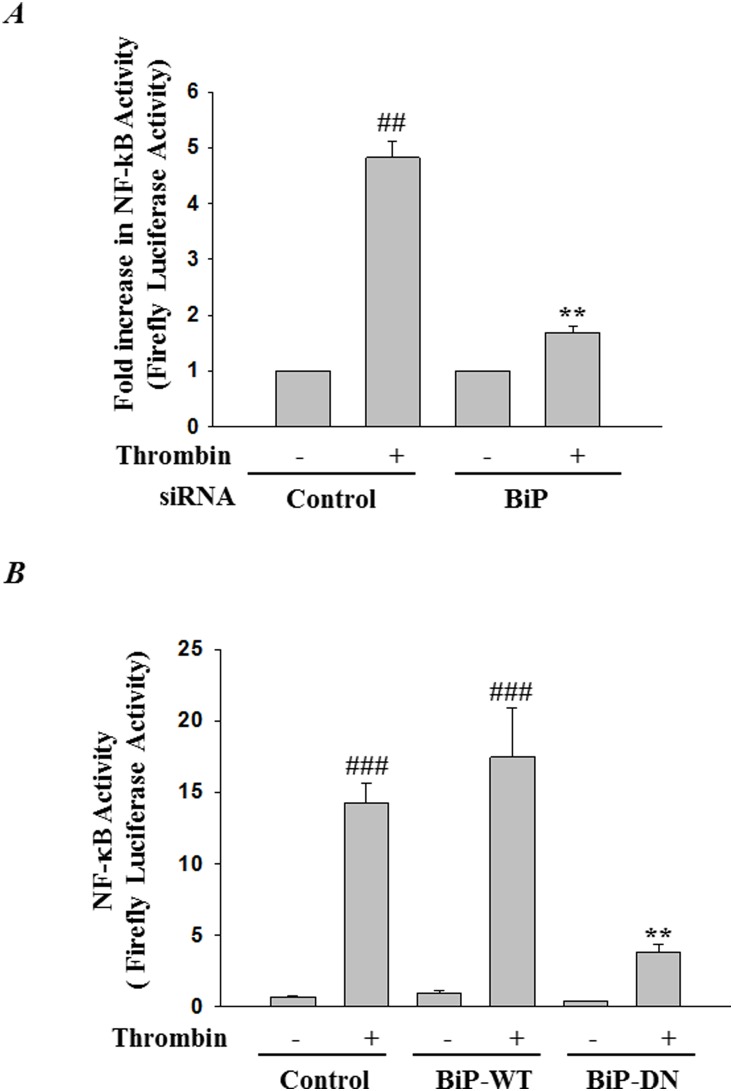
BiP knockdown attenuates thrombin-induced NF-κB reporter activity. HPAEC were transfected with control siRNA or BiP siRNA by use of DharmaFect1. Twenty-four hours later, cells were again transfected with NF-κBLUC construct by using DEAE-dextran as described in Materials and Methods. Cells were then challenged with *(* ***A*** *)* thrombin (5 U/ml) for 6 h, and the cell extracts were prepared and assayed for firefly and Renilla luciferase activities. The data were expressed as a fold increase in firefly to Renilla luciferase activities. Data are means ± SE (n = 4–6 for each condition). ^##^ *p*<0.01 difference from controls; **p<0.01 difference from thrombin stimulated controls. *(* ***B*** *)* HPAEC were transfected with NF-κBLUC in combination with BiP WT (pCMV-BiP-Myc-KDEL-WT) or BiP dominant negative (pCMV-BiP-Myc-KDEL-T37G) as described in Materials and Methods. Sixteen hours after transfection cells were treated with thrombin (5 U/ml) for 6 hrs. Cell extracts were prepared and assayed for firefly and Renilla luciferase activities. The data were expressed as a ratio of firefly to Renilla luciferase activities. Data are means ± SE (n = 4–6 for each condition). ^###^ *p*<0.001 difference from controls; **p<0.01 difference from thrombin stimulated controls.

**Figure 3:**
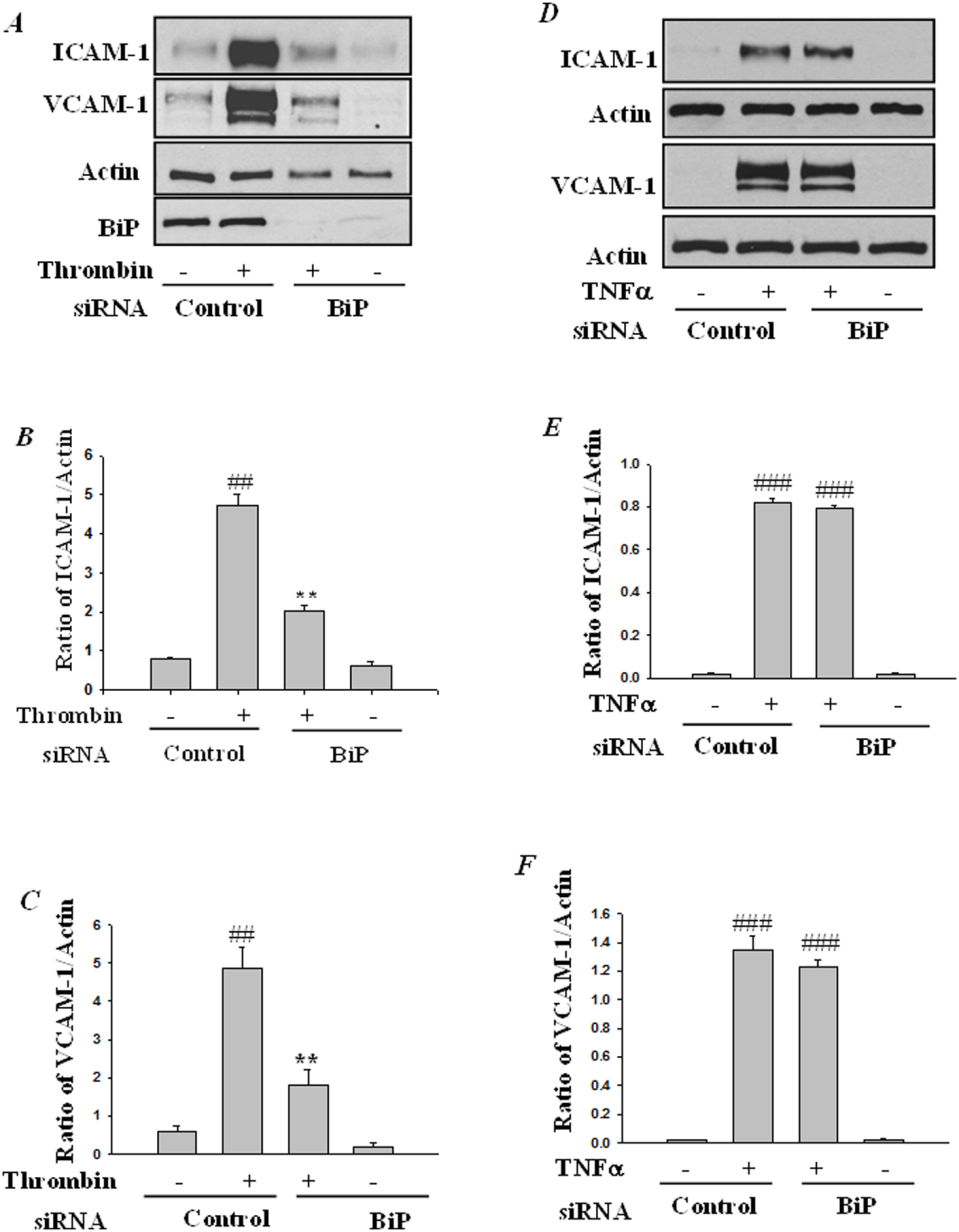
BiP knockdown mitigates thrombin-induced adhesion molecule expression. HPAEC were transfected with control siRNA or BiP-siRNA using DharmaFect1. After 24–36 h, the cells were challenged with *(* ***A*** *)* thrombin (5 U/ml) or *(* ***D*** *)* TNFα (100 U/ml), for 6 h. Total cell lysates were immunoblotted with an anti-ICAM-1, anti VCAM-1, and anti-BiP antibody. Actin was used to monitor loading. The bar graph represents the effect of BiP depletion on *(* ***B & C*** *)* thrombin and *(* ***E & F*** *)* TNFα-induced ICAM-1 and VCAM-1 expression normalized to actin level. The data are the means ± S.E. (n = 6 for each condition). ^##^ *p*<0.01 or ^###^ *p*<0.001 difference from controls; **p<0.01 difference from thrombin stimulated controls.

Next, to determine the role of BiP-mediated ER stress in EC inflammation, we monitored the effect of BiP knockdown on the activity of NF-κB, the master regulator of inflammation. Cells were transfected with pNF-κB -LUC in combination with either control-siRNA or BiP-siRNA. Results showed that thrombin challenge of cells transfected with control-siRNA resulted in increased NF-κB reporter activity and this response was inhibited in cells transfected with BiP-siRNA (Fig. 2A). In a reciprocal approach we addressed the effect of overexpression of BiP wild-type and a BiP dominant negative mutant on thrombin-induced NF-κB activity. Transfection of cells with a construct encoding wild-type BiP (pCMV-BiP-Myc-KDEL-WT) showed no effect on thrombin-induced NF-κB activity. However, expression of a construct encoding a dominant negative BiP (pCMV-BiP-Myc-KDEL-T37G) significantly inhibited thrombin-induced NF-κB activity (Fig. 2B). In view of the essential role of NF-κB in adhesion molecule expression, we determined the effect of BiP knockdown on thrombin-induced expression of ICAM-1 and VCAM-1. We found that depletion of BiP attenuated thrombin-induced ICAM-1 and VCAM-1 expression consistent with its effect on NF-κB activity (Fig. 3A–C). To further ascertain our observation that ER stress inhibits NF-κB activity, cells were treated with a known inducer of ER stress, tunicamycin. Results (Figure S1) show that tunicamycin significantly inhibited thrombin-induced NF-κB activity and adhesion molecule expression. Additionally, to investigate the specificity of the above response, we determined whether knockdown of BiP affected TNFα activation of NF-κB-induced ICAM-1 and VCAM-1 expression. Results showed that depletion of BiP (Fig. 3D–F) failed to inhibit TNFα-induced ICAM-1 and VCAM-1 expression. Together, these results indicate that in HPAEC, BiP regulates adhesion molecules expression in a stimulus specific manner.

### RNAi knockdown of BiP interferes with IKKβ activation and subsequent degradation of IκBα

**Figure 4:**
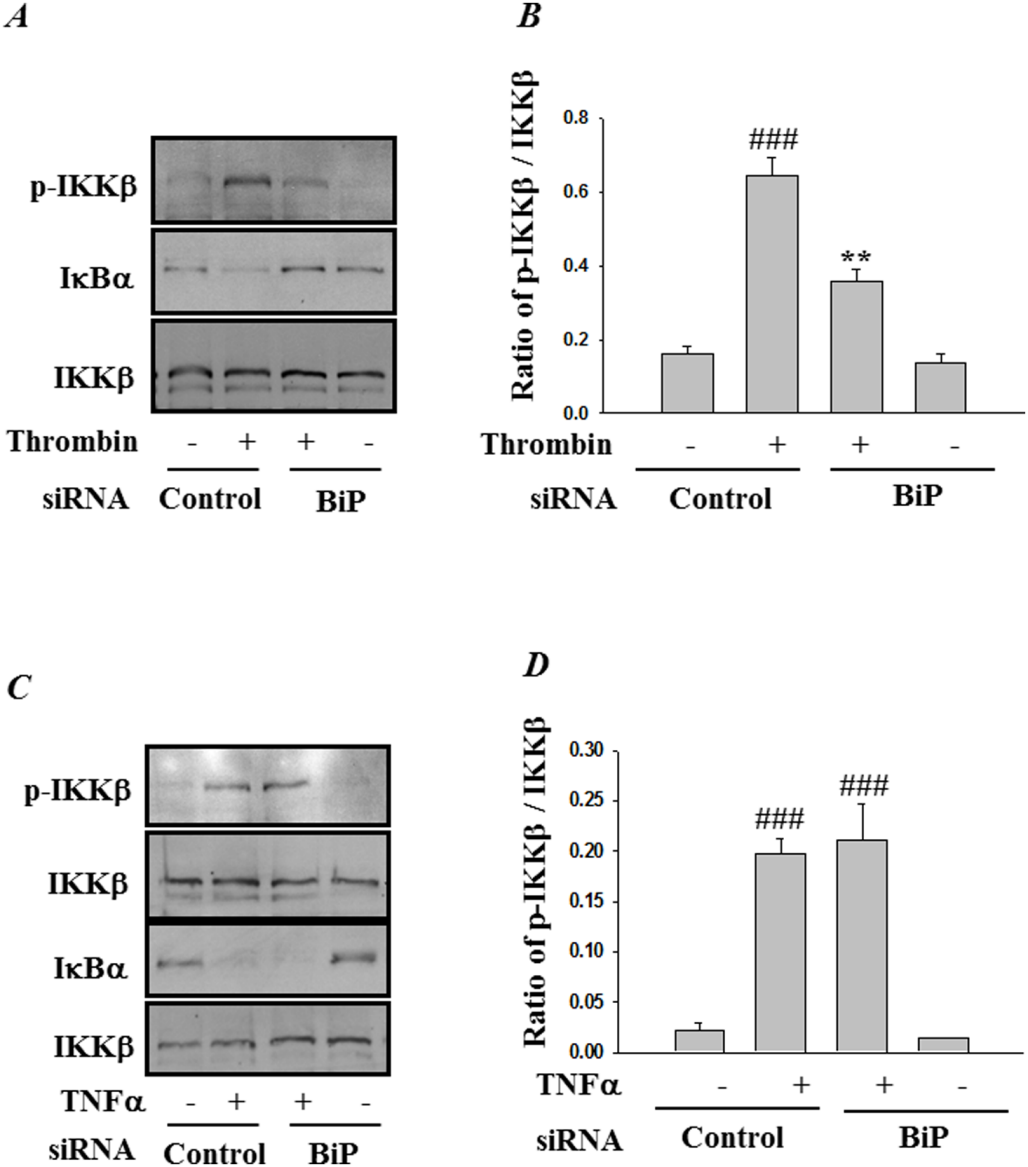
BiP knockdown blocks thrombin-induced IKKβ phosphorylation and IκBα degradation. HPAEC were transfected with control siRNA or BiP siRNA using DharmaFect1. After 24–36 h, the cells were challenged for 1 h with *(* ***A*** *)* thrombin (5 U/ml) or 0.5 h with *(* ***C*** *)* TNFα (100 U/ml). Total cell lysates were prepared and immunoblotted with anti-phospho-IKKβ and anti-IκBα to determine the phosphorylation of IKKβ and degradation of IκBα respectively. Total levels of IKKβ were used to monitor loading. The bar graphs represent the effect of BiP depletion on *(* ***B*** *)* thrombin-induced or *(* ***D*** *)* TNFα-induced IKKβ phosphorylation normalized to total IKKβ levels. The data are the means ± S.E. (n = 3–6 for each condition). ^###^ *p*<0.001 difference from controls; **p<0.01 difference from thrombin stimulated controls.

Next we analyzed the mechanism by which BiP depletion inhibits the NF-κB signaling cascade in endothelial cells. Phosphorylation of IκBα and its subsequent degradation is a requirement for the release and translocation of NF-κB to the nucleus. Since phosphorylation of IκBα is mediated by the IKK complex, we first evaluated the role BiP in IKK activation. Depletion of BiP inhibited activation of IKK upon thrombin challenge, as determined by decreased phosphorylation of IKKβ at Ser^176/181^ (Fig. 4A & B). We next examined whether knockdown of BiP affects IKKβ-mediated degradation of IκBα. Results show that depletion of BiP was effective in inhibiting IκBα degradation. In contrast, BiP depletion had no significant effect on TNFα-induced IKKβ phosphorylation and consequently on IκBα degradation (Fig. 4C & D).

### RNAi knockdown of BiP inhibits thrombin-induced NF-κB nuclear translocation and subsequent DNA binding

**Figure 5:**
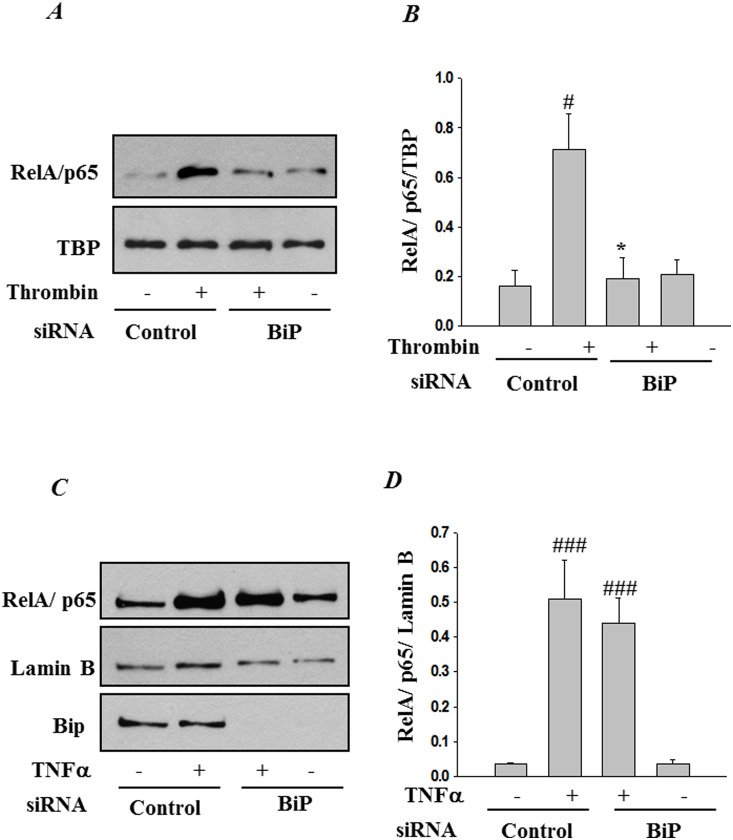
BiP knockdown prevents thrombin-induced RelA/p65 translocation to the nucleus. HPAEC were transfected with control-siRNA or BiP siRNA using DharmaFect1. After 24–36 h, the cells were challenged with *(* ***A*** *)* thrombin (5 U/ml) for 1 h or *(* ***C*** *)* TNFα for 0.5 h. Nuclear extracts (NE) were separated by SDS-PAGE and immunoblotted with anti-RelA/p65 antibody. Tata Binding Protein (TBP) and Lamin B, both nuclear proteins, were used as loading control for nuclear extracts. Anti-BiP antibody was used to monitor BiP depletion. The bar graph represents the effect of BiP knockdown on *(* ***B*** *)* thrombin or *(* ***D*** *)* TNFα-induced nuclear translocation of RelA/p65 normalized to TBP and Lamin B levels respectively. The data are the means ± S.E. (n = 6 for each condition). ^#^ *p*<0.05 or ^###^ *p*<0.001 difference from controls; *p<0.05 difference from thrombin stimulated controls.

**Figure 6:**
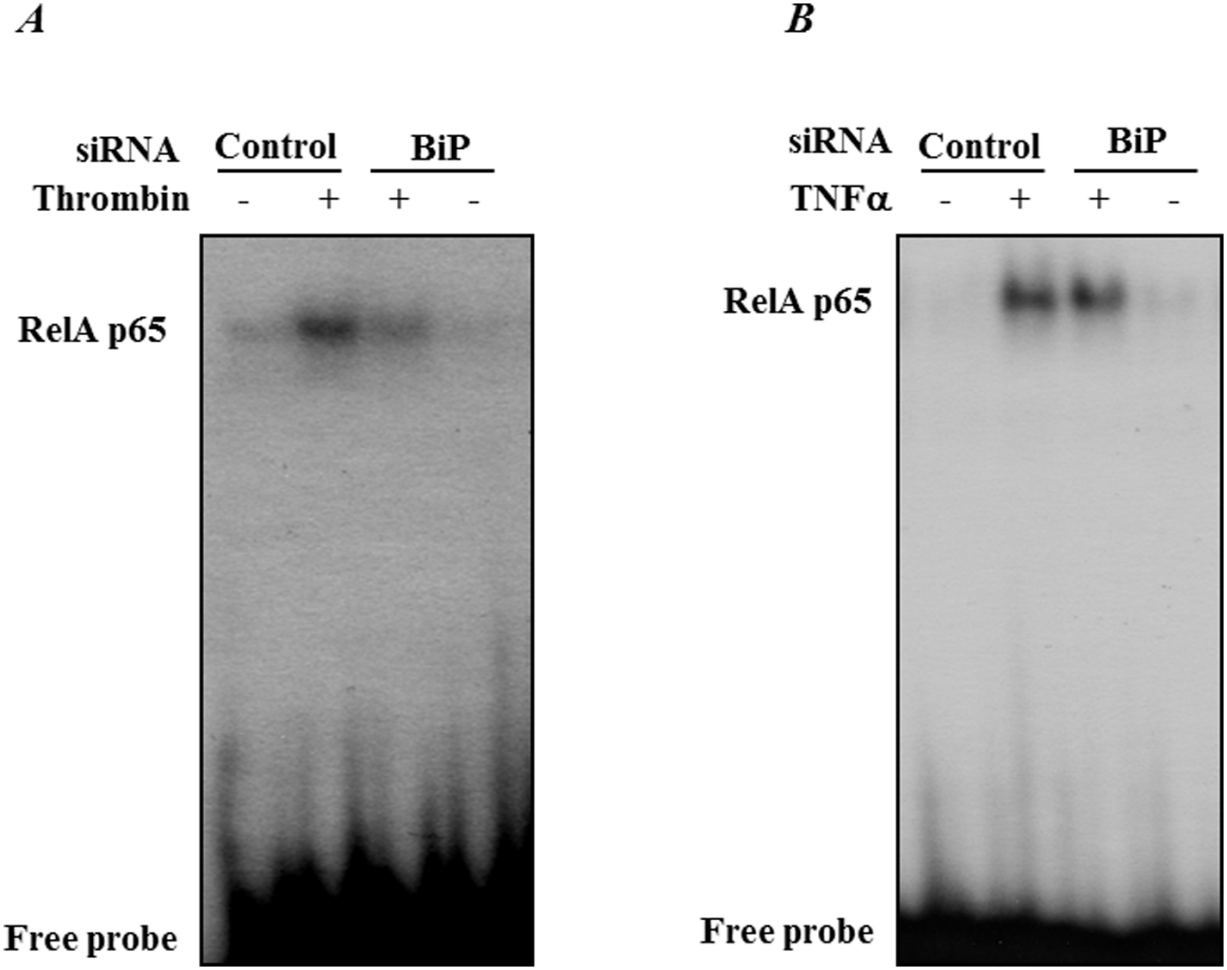
BiP knockdown blocks thrombin-induced RelA/p65 binding to DNA. HPAEC were transfected with control-siRNA or BiP-siRNA using DharmaFect1. After 24–36 h, the cells were challenged for 1 h with *(* ***A*** *)* thrombin or 0.5 h with *(* ***B*** *)* TNFα. Nuclear extracts were prepared and assayed for DNA binding of RelA/p65 by EMSA as described in the Materials and Methods. Results are representatives of two experiments.

Since IκBα degradation is a prerequisite for the release of NF-κB for its nuclear translocation, we next assessed the effect of BiP depletion on NF-κB nuclear translocation and subsequent DNA binding. Nuclear extracts from control and treated cells were analyzed by immunoblotting and electrophoretic mobility shift assay. Results showed a marked decrease in nuclear translocation (Fig. 5A & B) and DNA binding of RelA/p65 (Fig. 6A) in thrombin-treated cells transfected with BiP-siRNA, as compared to cells transfected with control-siRNA. Together these results are consistent with our previous data showing a block in IκBα degradation upon BiP depletion. Interestingly, results show that TNFα-induced RelA/p65 nuclear translocation (Fig. 5C & D) and subsequent DNA binding (Fig. 6B) were unchanged upon knockdown of BiP.

### RNAi knockdown of BiP regulates thrombin-induced NF-κB phosphorylation at Ser^536^

**Figure 7:**
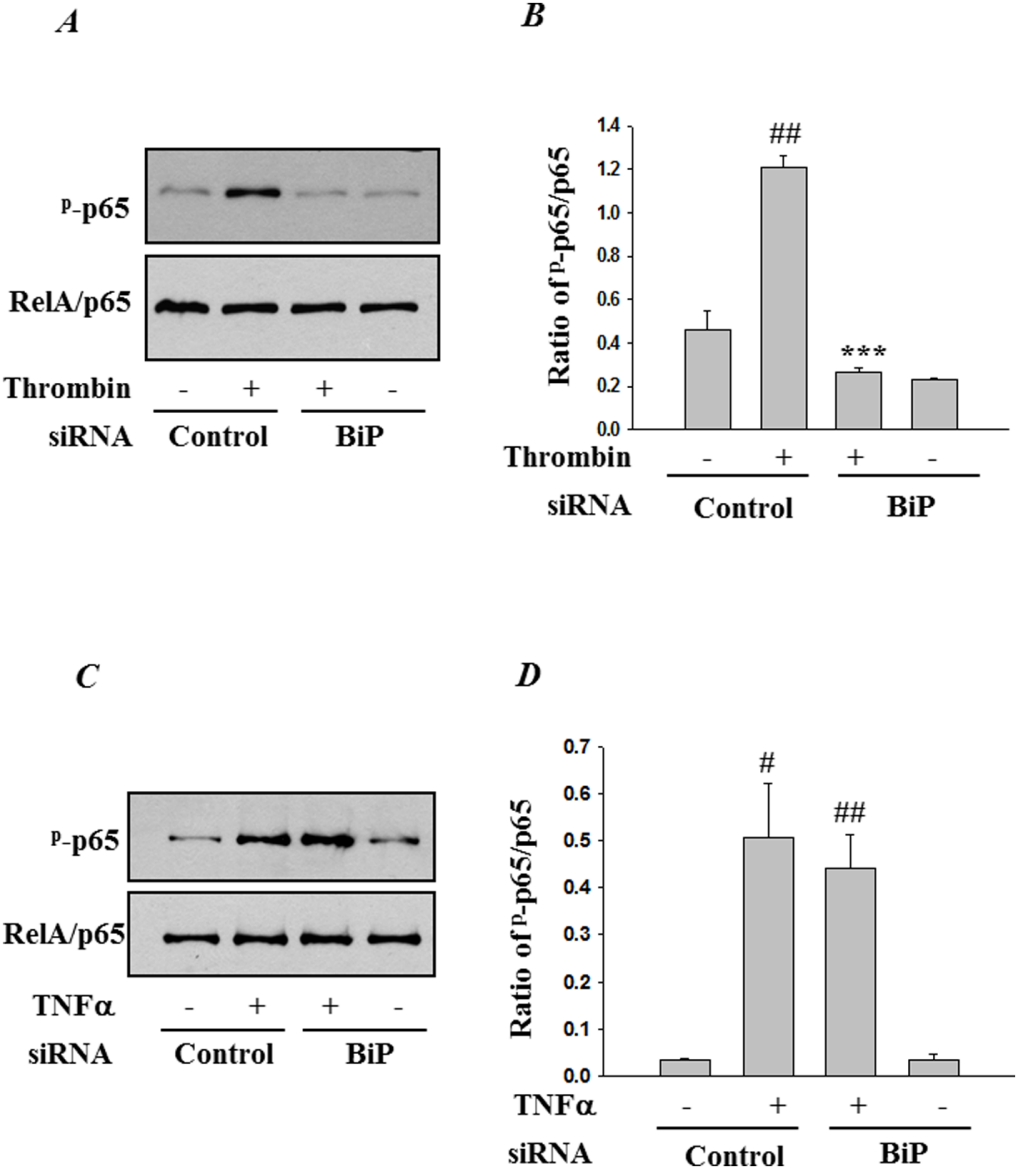
BiP knockdown markedly ameliorates thrombin-induced RelA/p65 phosphorylation at Ser^536^. HPAEC were transfected with control-siRNA or BiP-siRNA using DharmaFect1. After 24–36 h, the cells were challenged for 1 h with *(* ***A*** *)* thrombin or 0.5 h with *(* ***C*** *)* TNFα. Cell lysates were immunoblotted with an anti-phospho RelA/p65 (Ser^536^) to determine the phosphorylation status of RelA/p65. The levels of total RelA/p65 were used to monitor loading. The bar graph represents the effect of BiP depletion on *(* ***B*** *)* thrombin or *(* ***D*** *)* TNFα-induced phosphorylation of RelA/p65 at Ser^536^ normalized to total RelA/p65 levels. The data are the means ± S.E. (n = 3 for each condition). ^#^ *p*<0.05 or ^##^ *p*<0.01 difference from controls; ***p<0.001 difference from thrombin stimulated controls.

We also determined whether BiP contributes to the transactivating potential of RelA/p65, in addition to promoting IκBα degradation, nuclear translocation and DNA binding function. To this end, BiP-depleted cells were challenged with thrombin and total lysates were analyzed for Ser^536^ phosphorylation of RelA/p65. Depletion of BiP caused a significant inhibition in RelA/p65 phosphorylation caused by thrombin (Fig. 7A & B). As expected, TNFα-induced RelA/p65 phosphorylation remained unaffected (Fig. 7C & D), further establishing the notion that BiP mediates EC inflammation in a stimulus-specific manner.

### RNAi knockdown of BiP promotes filamentous actin formation and abrogates endothelial permeability

**Figure 8:**
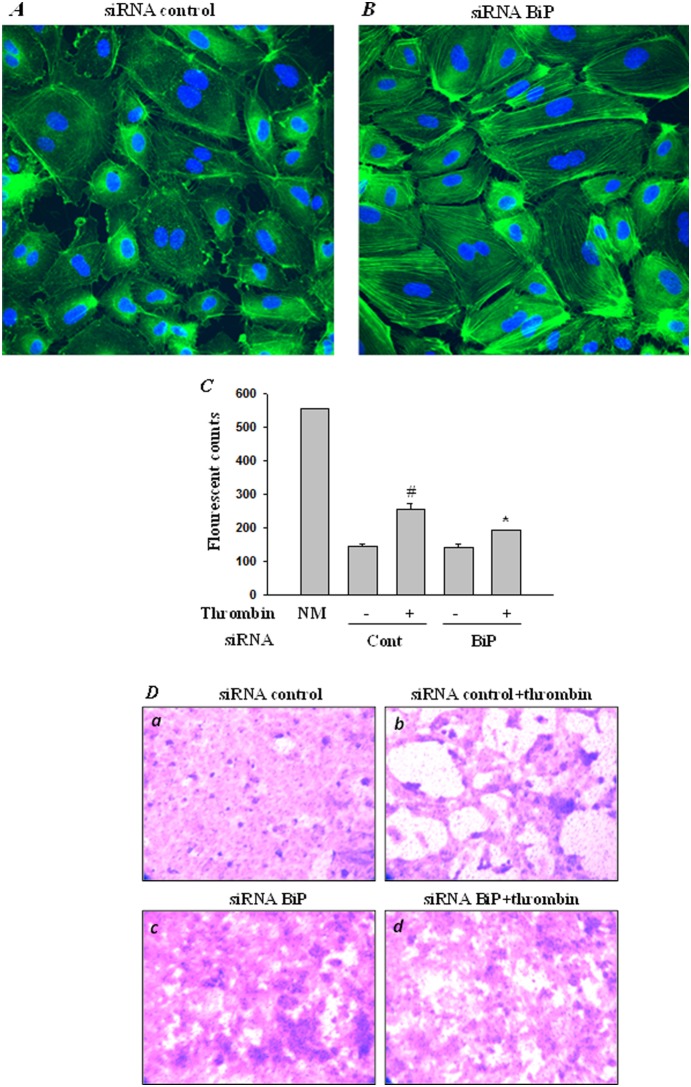
BiP knockdown potentiates actn filment formation and regulates thrombin-induced endothelial permeability. HPAEC were transfected with *(* ***A*** *)* control-siRNA or *(* ***B*** *)* BiP-siRNA using DharmaFect1. After 24–36 h cells were fixed, permeabilized, and stained with Alexa Fluor 488 labeled phalloidin to visualize the actin filaments. Images were analyzed by Fluorescence microscopy. Results are representative of three experiments. *(* ***C*** *)* HPAEC transfected with control-siRNA or BiP- siRNA were seeded at 20,000 cells per transwell insert and cultured for 48 hours. Following this, the confluent monolayer was treated with thrombin (5 U/ml) for 30 minutes. FITC-Dextran permeability testing was done to check monolayer integrity. Permeation was stopped by removing the inserts from the wells. Media from the receiver tray was transferred to a 96 well opaque plate to measure fluorescence. Fluorescent intensities were quantified using a fluorescent plate reader with filters appropriate for 485 nm and 535 nm excitation and emission. The data are the means ± S.E. (n = 3–6 for each condition). ^#^ *p*<0.05 difference from controls; *p<0.05 difference from thrombin-stimulated controls. *(* ***D*** *)* Following permeability testing the endothelial monolayer transfected with control siRNA (a & b) or with BiP siRNA (c & d), followed by treatment with thrombin (b & d) or left untreated (a & c) were stained for bright field imaging.

Previous work in our lab has shown that the actin cytoskeleton is a dynamic structure that undergoes rearrangement upon exposure to thrombin. Thrombin engages actin depolymerizing protein cofilin, as well as cofilin kinase LIMK1 and cofilin phosphatase SSH-1L to regulate actin dynamics [38], [40] in endothelial cells. The endothelial cell cytoskeleton is primarily composed of three structures: actin microfilaments, microtubules and intermediate filaments. It is the actin filaments that are of critical importance to EC permeability and their role is much more defined as compared to microtubules and intermediate filaments [46]. These studies prompted us to assess the role of BiP in regulating actin cytoskeletal dynamics and endothelial barrier integrity leading to vascular permeability. Controls as well as BiP-depleted cells were stained with Alexa Flour 488-labeled phalloidin to visualize actin filaments. Results show an increase in filamentous actin formation in cells depleted of BiP as compared to control cells (Fig. 8A & B). Furthermore the effect of actin cytoskeleton rearrangement on endothelial permeability was analyzed using an *in vitro* permeability assay. EC permeability is mostly regulated by intercellular junction organization. The intercellular boundaries in confluent resting cells are maintained by adherence-junction proteins such as VE-cadherin and catenin. Thrombin a serine protease, acts by cleaving its receptor PAR1 causing an increase in intracellular calcium and PKC activation, this in turn stimulates the endothelial contractile apparatus. The endothelial contraction results in disruption of VE-cadherin/catenin complex leading to intercellular gap formation. This effect of thrombin is reversible and is responsible for increased endothelial permeability [47]. To, this end HPAEC transfected with BiP-siRNA or control-siRNA were seeded at 20,000 cells per well into transwell inserts containing 1 µm pores within a transparent polyethylene terephthalate (PET) membrane coated with type 1 rat-tail collagen, and cultured for 48 hours. The confluent monolayer was then treated with thrombin (5 U/ml) for 30 minutes, followed by addition of a high molecular weight FITC-Dextran on top of the cells. The movement of the fluorescent molecule across the endothelial monolayer is a direct determinant of monolayer permeability. Our results show that knockdown of BiP significantly reduced thrombin-induced permeability, as indicated by a marked decrease in the fluorescent counts measured in the receiver tray using a fluorescent plate reader (Fig. 8C). Next the endothelial monolayer was stained to monitor monolayer integrity using Cell stain provided in the kit. Data show that knockdown of BiP significantly reduced the gaps between untreated and thrombin treated cells, implying the role of BiP in EC integrity (Fig. 8D).

### Inactivation of BiP by Subtilase Cytotoxin (SubAB) induces ER stress

**Figure 9:**
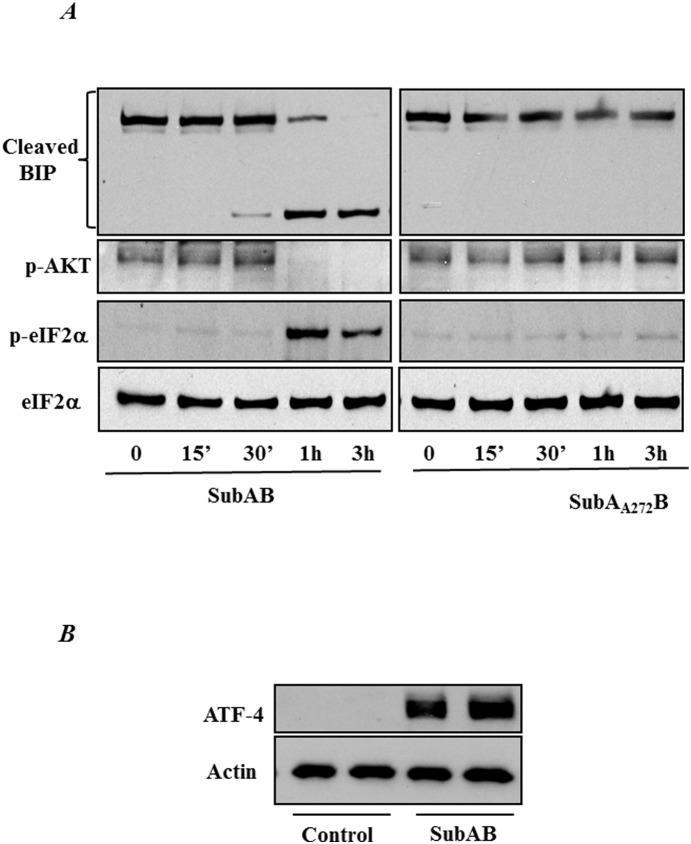
SubAB induces BiP cleavage and ER stress. *(* ***A*** *)* HPAEC were treated with 0.1 µg/ml of SubAB or mutant SubA_A272_B for the indicated time points. Cell lysates were immunoblotted with an anti-phospho eIF2α antibody and anti-phospho- AKT antibody to determine the induction of ER stress. The levels of total eIF2α were used to monitor loading. BiP antibody was used to monitor the cleavage of BiP by SubAB. *(* ***B*** *)* HPAECs were treated with 0.1 µg SubAB for 3 hours and cell lysates were immunoblotted for ATF-4 antibody. Actin was used as a loading control.

In order to further ascertain the role of BiP in EC inflammation and injury we used an alternative approach. Cells were treated with SubAB, the prototype of a family of AB_5_ cytotoxins produced by Shiga toxigenic *Escherichia coli* that specifically cleaves and inactivates BiP, resulting in the activation ER stress and therefore UPR [48]. A time course experiment showed that treatment of HPAEC with SubAB induced BiP cleavage at 1 hour, which coincided with the phosphorylation of eIF2α. We also observed a decrease in AKT-phosphorylation at 1 hour of SubAB treatment. In contrast, treatment of cells with SubA_A272_B, a nontoxic variant of SubAB, failed to cleave BiP, induce eIF2α phosphorylation and decrease AKT-phosphorylation (Fig. 9A). As expected the level of ATF4 was also induced upon SubAB treatment (Fig. 9B). These data indicate that SubAB specifically cleaves BiP and thereby induce ER stress.

### Preconditioning HPAEC with SubAB-induced ER stress suppressed NF-κB transcriptional activity and proinflammatory gene expression

**Figure 10:**
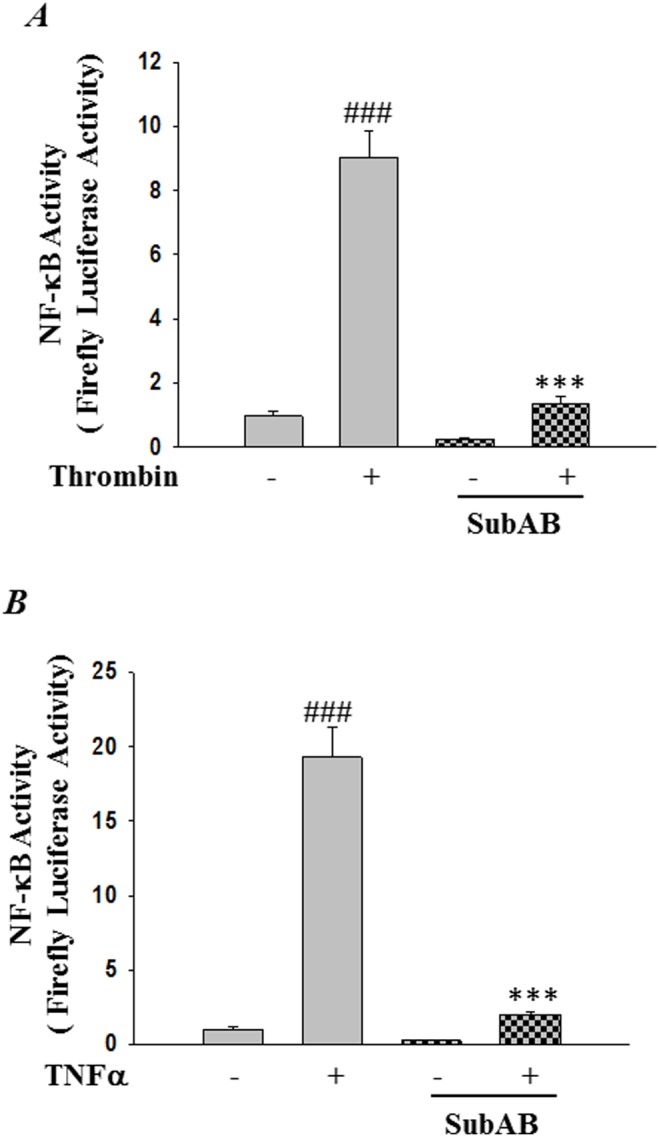
SubAB inhibits NF-κB transcriptional activity. HPAEC were transfected with NF-κBLUC construct by using DEAE-dextran as described in Materials and Methods. After 24 hours cells were treated with 0.1 µg/ml of SubAB or mutant SubA_A272_B for 6 h, followed by treatment with *(* ***A*** *)* thrombin (5 U/ml) or *(* ***B*** *)* TNFα (100 U/ml) for 6 h. Cell extracts were prepared and assayed for Firefly and Renilla luciferase activities. The data were expressed as a ratio of Firefly to Renilla luciferase activities. Data are means ± SE (n = 4–6 for each condition). ^###^ *p*<0.001 difference from controls; ***p<0.001 difference from thrombin and TNF-α stimulated controls.

**Figure 11:**
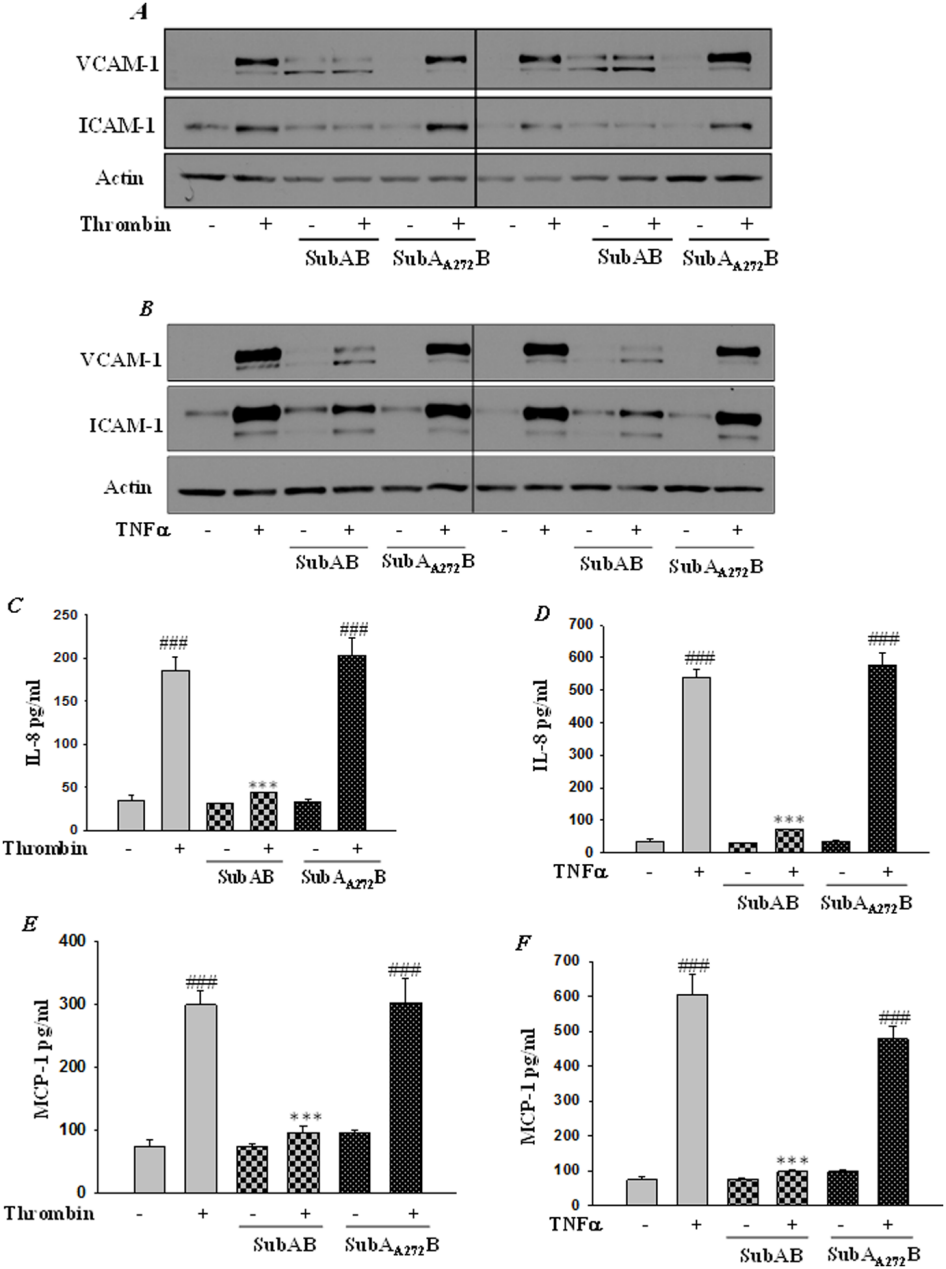
SubAB inhibits NF-κB mediated proinflammatory gene expression. HPAEC were treated with 0.1 µg/ml of SubAB or mutant SubA_A272_B for 6 h, followed by treatment with *(* ***A & C & E*** *)* thrombin (5 U/ml) or *(* ***B & D & F*** *)* TNFα (100 U/ml) for 6 h. Total cell lysates were immunoblotted with *(* ***A & B*** *)* anti-ICAM-1 antibody and anti -VCAM-1 antibody. Actin was used to monitor loading**.** The conditioned media were subjected to ELISA to determine the levels of *(* ***C & D*** *)* IL-8 or *(* ***E & F*** *)* MCP-1. Data are means ± S.E. (n = 6–9 for each condition). ^###^ *p*<0.001 difference from control; ***p<0.001 difference from thrombin and TNFα-stimulated controls.

Expression of adhesion molecules, cytokines and chemokines in endothelial cells by inflammatory mediators such as thrombin and TNFα is a central step in the pathogenesis of ALI and is under the tight regulation of the transcription factor NF-κB. We determined whether SubAB-mediated ER stress preconditioning affects thrombin and TNFα-induced NF-κB activation and subsequent expression of adhesion molecules ICAM-1, VCAM-1, and chemo-attractant proteins IL-8 and MCP-1. Cells were transfected with pNF-κB-LUC for 24 hours, followed by treatment with SubAB for 6 hours. Results showed that thrombin or TNFα challenge of cells resulted in increased NF-κB reporter activity and this response was inhibited in cells pretreated with 0.1 µg/ml of SubAB (Fig. 10A & B). Next we determined the effect of SubAB on NF-κB target genes, ICAM-1 and VCAM-1. HPAEC were exposed to 0.1 µg/ml SubAB and SubA_A272_B for 6 hours and then treated with 5 U/ml thrombin and 100 U/ml of TNFα for 6 hours. Western blot analysis using total cell lysates showed a reduced expression of both thrombin- and TNFα-induced ICAM-1 and VCAM-1 expression, in cells treated with SubAB (Fig. 11A & B). ELISA using culture supernatants showed a marked reduction in the levels of thrombin- and TNFα-induced for IL-8 and MCP-1, in cells preconditioned with SubAB (Fig. 11C–F). In contrast, preconditioning of HPAECs to SubA_A272_B, did not affect any of the above responses (Fig. 11C–F).

### SubAB attenuates EC inflammation independent of IκBα degradation and NF-κB nuclear translocation

**Figure 12:**
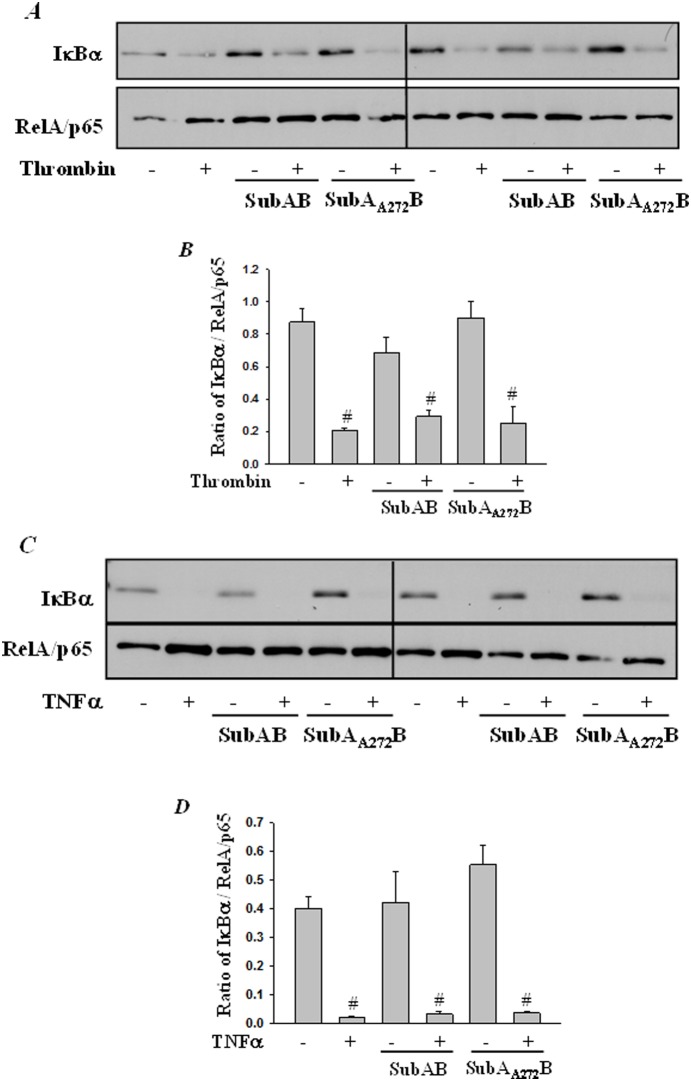
SubAB failed to block thrombin-induced IκBα degradation. HPAEC were treated with 0.1 µg/ml of SubAB or mutant SubA_A272_B for 6 h, followed by treatment with *(* ***A*** *)* thrombin (5 U/ml) for 1 h or with *(* ***C*** *)* TNFα (100 U/ml) for 30 min. Total cell lysates were prepared and immunoblotted with anti-IκBα antibody to determine degradation of IκBα. Levels of RelA/p65 was used to monitor loading. The bar graphs represent the effect SubAB and SuBA_A272_B on *(* ***B*** *)* thrombin-induced or *(* ***D*** *)* TNFα-induced IκBα degradation normalized to total RelA/p65 levels. The data are the means ± S.E. (n = 3–6 for each condition). ^#^ *p*<0.05 difference from controls.

**Figure 13:**
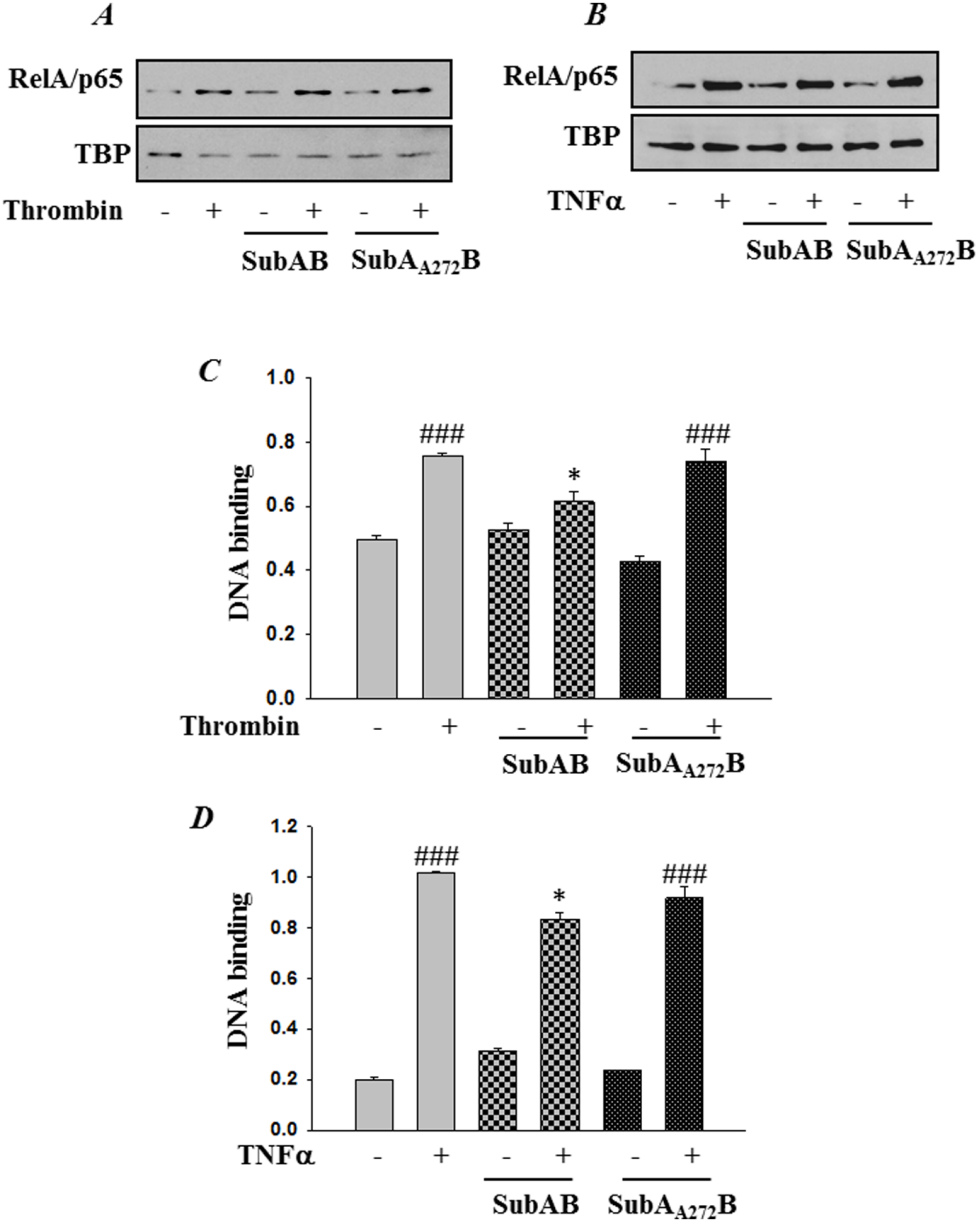
SubAB attenuates RelA/p65 DNA binding independent of its nuclear translocation. HPAEC were treated with 0.1 µg/ml of SubAB or mutant SubA_A272_B for 6 h, followed by treatment with *(* ***A & C*** *)* thrombin (5 U/ml) for 1 h or with *(* ***B & D*** *)* TNFα (100 U/ml) for 30 min. *(* ***A & B*** *)* Nuclear extracts (NE) were separated by SDS-PAGE and immunoblotted with anti-RelA/p65 antibody. Tata Binding Protein (TBP) was used as a loading control for nuclear extract. *(* ***C & D*** *)* Nuclear extracts were assayed for DNA binding of RelA/p65 by Cayman’s NF-κB (RelA/p65) Transcription Factor Assay Kit as described in Materials and Methods. The data are the means ± S.E. (n = 6 for each condition). ^###^ *p*<0.001 difference from controls; *p<0.05 difference from thrombin and TNF-α stimulated controls.

Next we determined whether the reduced NF-κB activity and subsequent adhesion molecule and chemokine expression following preconditioning of HPAEC with SubAB is due to reduced degradation of IκBα, the cytoplasmic inhibitor that sequesters NF-κB in the cytosol and thereby blocks its translocation to the nucleus and consequently gene transcription. Interestingly, analysis of the cytoplasmic extracts showed that preconditioning of cells with SubAB did not inhibit thrombin or TNFα-induced IαBα degradation (Fig. 12A–D). Furthermore, our data showed that the nuclear translocation of the liberated NF-κB also remained unaffected upon preconditioning with ER stress (Fig. 13A & B).

### Preconditioning cells with SubAB-induced ER stress suppressed NF-κB binding to DNA

Next we analyzed whether the reduced NF-κB activity and gene expression is due to inhibition of NF-κB DNA binding activity. To this end we used a nonradioactive, ELISA-based assay to monitor RelA/p65 binding to DNA in nuclear extracts. Results show that both thrombin and TNFα-induced binding of NF-κB to the DNA was significantly inhibited in cells pretreated with the ER stress inducer SubAB (Fig. 13C & D). However the treatment of cells with SubA_A272_B did not result in attenuation of RelA/p65 binding to DNA (Fig. 13C & D). Together these observations indicate that different ER stress inducers, RNAi-mediated knockdown of BiP versus SubAB-mediated cleavage of BiP, adopt different mechanisms to inhibit EC inflammation.

### Preconditioning cells with SubAB-induced ER stress potentiated F-actin formation and abrogated endothelial permeability

**Figure 14:**
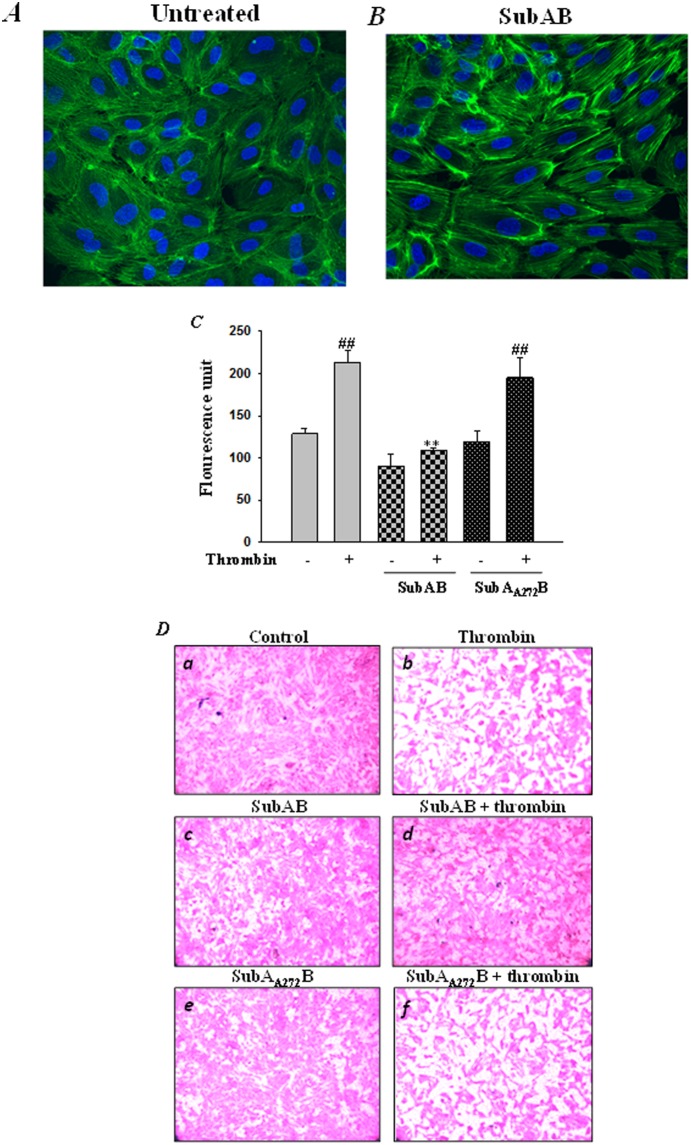
SubAB potentiates actin filament formation and regulates thrombin-induced endothelial permeability. HPAEC were left untreated *(* ***A*** *)* or *(* ***B*** *)* treated with 0.1 µg/ml of SubAB for 6 hours. The cells were then fixed, permeabilized, and stained with Alexa Fluor 488 labeled phalloidin to visualize the actin filaments. Images were analyzed by Fluorescence microscopy. Results are representative of three experiments. *(* ***C*** *)* HPAEC treated with 0.1 µg/ml of SubAB or mutant SubA_A272_B for 6 h were seeded at 20,000 cells per transwell insert and cultured for 48 hours. Following this, the confluent monolayer was treated with thrombin (5 U/ml) for 30 minutes. FITC-Dextran permeability testing was done to check monolayer integrity. Permeation was stopped by removing the inserts from the wells. Media from the receiver tray was transferred to a 96 well opaque plate to measure fluorescence. Fluorescent intensities were quantified using a fluorescent plate reader with filters appropriate for 485 nm and 535 nm excitation and emission. The data are the means ± S.E. (n = 3–6 for each condition). ^##^ *p*<0.01 difference from controls; **p<0.01 difference from thrombin stimulated controls. *(* ***D*** *)* Following permeability testing the endothelial monolayer representing various experimental conditions (as indicated in the Figure) was stained for bright field imaging.

Since SubAB-mediated preconditioning to ER stress mitigates thrombin- and TNFα- induced NF-κB activation, we next analyzed the effect of SubAB on actin dynamics and endothelial permeability, another salient feature contributing to the pathogenesis of ALI and ARDS. Our results show that similar to siRNA-mediated knockdown of BiP, SubAB-mediated inactivation of BiP also resulted in alterations in actin dynamics, as evidenced by an increase in Alexa Fluor 488-labeled phalloidin staining of actin stress fibers in SubAB treated cells compared to untreated cells (Fig. 14A & B). Next, in order to analyze whether SubAB-mediated alteration in actin dynamics affects endothelial permeability, we used an in vitro vascular permeability assay. Our data showed a significant inhibition in thrombin-induced permeability in cells pretreated with SubAB, as indicated by a decrease in fluorescence units. However, cells pretreated with the inactive mutant SubA_A272_B were unable to restore thrombin-induced endothelial permeability (Fig. 14C). In addition, monolayer integrity was visualized using Cell stain provided in the kit, under bright field microscopy. Cells pretreated with SubAB showed tightening of thrombin-induced intercellular gaps as compared to cells pretreated with the inactive SubAB mutant (Fig. 14D). Together these results imply that BiP is a central regulator of EC inflammation and injury.

## Discussion

Studies have shown that BiP, a central regulator of ER stress, is critically involved in the pathogenesis of cancer [49], and inflammation associated with rheumatoid arthritis [50] and diabetic retinopathy [51]. The focus of this study is to understand the relevance of ER stress in endothelial cell inflammation and injury associated with pulmonary inflammatory diseases such as ALI and ARDS. To begin with, the present study demonstrates that depletion or inactivation of BiP per se induced ER stress, as evidenced by increase in phosphorylation of the eukaryotic initiation factor alpha (eIF2α), and an increase in the expression of activated transcription factor 4 (ATF4), the two markers of ER stress. Next, our results indicate that preconditioning of endothelial cells with ER stress is protective against EC inflammation and injury. Mechanistic analysis revealed that the protective effect of ER stress is mediated via dampening of NF-κB activation and restoration of endothelial barrier dysfunction.

Recent studies have shown a protective effect of ER stress preconditioning against cytokine-induced inflammation in retinal endothelial and renal mesengial cells by suppressing NF-κB-mediated adhesion molecule expression [25], [28]. Also, pretreatment of rats with subnephritogenic doses of ER stress inducers tunicamycin or thapsigargin ameliorated mesangioproliferative glomerulonephritis [25]. These studies suggest a protective role of signaling pathways activated by ER stress, against inflammatory conditions, but the mechanisms are largely unknown. Our data show that siRNA mediated knockdown of BiP inhibited EC inflammation in a stimulus specific manner. Endothelial inflammation induced by thrombin, whose levels are elevated in the BALF of patients suffering from ALI [52], [53], is abolished upon BiP depletion as noted by inhibition of IKKβ-mediated phosphorylation and degradation of IκBα, an event responsible for the release and subsequent activation of NF-κB. Downstream NF-κB signaling events involving its cytoplasmic trafficking, phosphorylation at Ser^536^, DNA binding and resulting gene expression were consequently abrogated. Surprisingly, TNFα-induced activation of NF-κB and EC inflammation was not hindered upon BiP knockdown in HPAEC, indicating a stimulus-specific regulation of endothelial NF-κB by BiP. Interestingly, Zhang *et al.* show that tunicamycin –induced ER stress preconditioning abolished TNFα-elicited NF-κB activation and adhesion molecule expression via activation of XBP-1 in primary retinal microvascular endothelial cells (HREC) [28]. To understand the discrepancy we evaluated the role of XBP-1 in TNFα induced signaling in HPAEC by knocking down XBP-1 expression using siRNA. Results show that depletion of XBP-1 did not inhibit TNFα-induced adhesion molecule expression in HPAEC (data not shown). This disparity in cellular response can either be attributed to different ER stress inducers i.e., BiP-siRNA versus tunicamycin, or to endothelial heterogeneity i.e., HPAEC versus HREC. Studies have shown that endothelial cells from different size vessels or from same vessel but different sites show significant heterogeneity in structure and function [54].

In order to further substantiate our data we used an unrelated methodological approach. HPAEC were pretreated with sub toxic dose of SubAB, a serine protease characterized by a conserved catalytic triad Asp-His-Ser_272,_ which selectively cleaves BiP between a dileucine motif (Leu _416/417_), resulting in disruption of BiP function and thereby induction of ER stress [55]. An inactive mutant SubA_A272_B, in which Ser_272_ was mutated to an alanine, was used as a negative control. Our results show that unlike BiP knockdown-mediated abrogation of EC inflammation via inhibition of IκBα degradation, SubAB repressed EC inflammation by blocking the binding of the released NF-κB to the DNA and subsequent adhesion molecule, chemokine and cytokine expression. The events upstream of DNA binding such as IκBα degradation and nuclear translocation remain unaffected. As expected, the inactive SubA_A272_B failed to inhibit the above responses. These observations indicate that the two approaches resulted in a similar outcome; inhibition of EC inflammation, however via different mechanism, which can be biologically informative and therapeutically relevant. Earlier studies have shown that the inhibition of Aurora Kinases and PI3K using siRNA-mediated gene silencing or pharmacological inhibitor-mediated inactivation resulted in phenotypes that were not consistent [56]. Kitamura *et al.*, have also shown a protective effect of SubAB on TNFα-induced inflammation in rat epithelial cells via induction of C/EBPβ [27]. SubAB has also been shown to protect mice against LPS-induced experimental arthritis [57]. Furthermore, our studies show that BiP plays a central role in EC injury via regulation of endothelial barrier integrity. Preconditioning EC with ER stress by knockdown or inactivation of BiP significantly blocked thrombin-induced endothelial permeability.

In summary our studies demonstrate that the dual mechanism by which BiP regulates EC inflammation involves the activation of the NF-κB pathway and disruption of endothelial barrier integrity. Thus, the specific targeting of BiP may be a strategy for dampening inflammatory responses associated with intravascular coagulation and sepsis in mice.

## Supporting Information

Figure S1 ***(A)*** **Tunicamycin attenuates thrombin-induced NF-κB reporter activity.** HPAEC were transfected with NF-κBLUC and Renilla luciferase construct by using DEAE-dextran as described in Materials and Methods. Cells were then treated with 0.5 µg/ml tunicamycin for 30 minutes followed by challenge with thrombin (5 U/ml) for 6 hours. Cell extracts were prepared and assayed for firefly and Renilla luciferase activities. The data were expressed as a ratio of firefly to Renilla luciferase activities. Data are means ± SE (n = 4–6 for each condition). ^###^ *p*<0.001 difference from controls; ***p<0.001 difference from thrombin stimulated controls. ***(B)*** **Tunicamycin inhibits thrombin-induced adhesion molecule expression.** HPAEC were treated with 0.5 µg/ml tunicamycin for 30 minutes followed by challenge with thrombin (5 U/ml) for 6 hours. Total cell lysates were immunoblotted with an anti-ICAM-1, anti VCAM-1, and anti-BiP antibody. Actin was used to monitor loading. The bar graphs represent the effect of tunicamycin on thrombin-induced ***(C)*** ICAM-1 and ***(D)*** VCAM-1 expression normalized to actin level. The data are the means ± S.E. (n = 6 for each condition). ^##^ *p*<0.01 or ^###^ *p*<0.001 difference from controls; ***p<0.001 difference from thrombin stimulated controls.
(TIF)
