# Supplementary figures and images for: Preconditioning with Endoplasmic Reticulum Stress Ameliorates Endothelial Cell Inflammation
Source: PLoS One. 2014 Oct 30;9(10):e110949. doi: 10.1371/journal.pone.0110949 (PMC4214695; doi:10.1371/journal.pone.0110949)

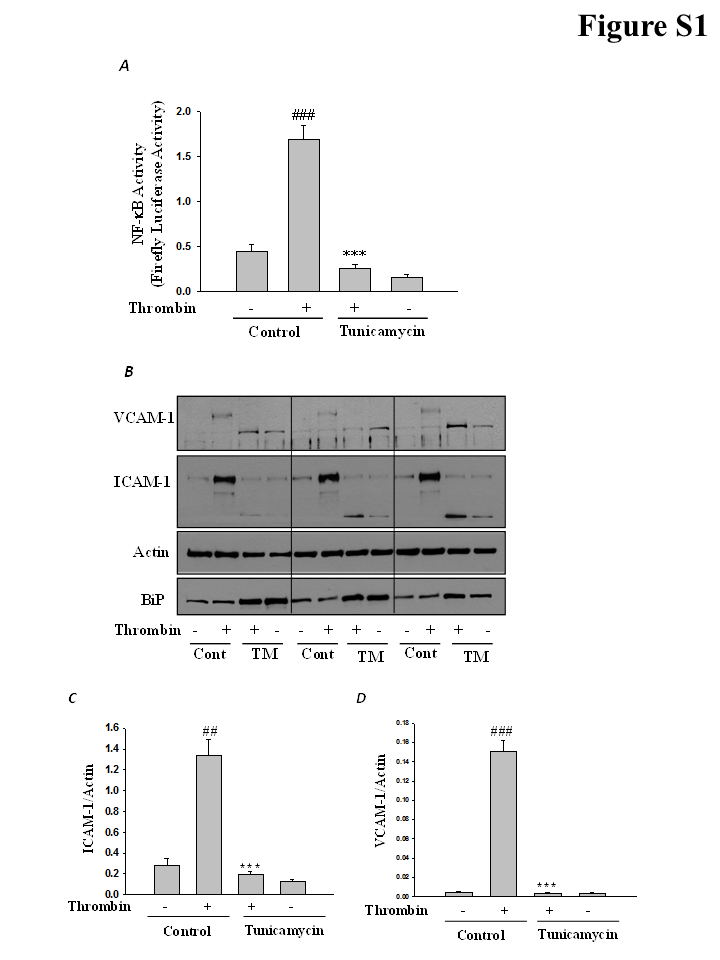

Supplement: Figure S1 — (A) Tunicamycin attenuates thrombin-induced NF-κB reporter activity. HPAEC were transfected with NF-κBLUC and Renilla luciferase construct by using DEAE-dextran as described in Materials and Methods. Cells were then treated with 0.5 µg/ml tunicamycin for 30 minutes followed by challenge with thrombin (5 U/ml) for 6 hours. Cell extracts were prepared and assayed for firefly and Renilla luciferase activities. The data were expressed as a ratio of firefly to Renilla luciferase activities. Data are means ± SE (n = 4–6 for each condition). ### p<0.001 difference from controls; ***p<0.001 difference from thrombin stimulated controls. (B) Tunicamycin inhibits thrombin-induced adhesion molecule expression. HPAEC were treated with 0.5 µg/ml tunicamycin for 30 minutes followed by challenge with thrombin (5 U/ml) for 6 hours. Total cell lysates were immunoblotted with an anti-ICAM-1, anti VCAM-1, and anti-BiP antibody. Actin was used to monitor loading. The bar graphs represent the effect of tunicamycin on thrombin-induced (C) ICAM-1 and (D) VCAM-1 expression normalized to actin level. The data are the means ± S.E. (n = 6 for each condition). ## p<0.01 or ### p<0.001 difference from controls; ***p<0.001 difference from thrombin stimulated controls. (TIF) [file pone.0110949.s001.tif]
